# Review of Cases of Angiostrongyliasis in Hawaii, 2007–2017

**DOI:** 10.4269/ajtmh.19-0280

**Published:** 2019-07-08

**Authors:** David I. Johnston, Marlena C. Dixon, Joe L. Elm, Precilia S. Calimlim, Rebecca H. Sciulli, Sarah Y. Park

**Affiliations:** Hawaii Department of Health, Honolulu, Hawaii

## Abstract

Angiostrongyliasis, caused by the *Angiostrongylus cantonensis* roundworm, became reportable in the state of Hawaii in 2007. We confirmed 82 reported cases between 2007 and 2017. There was a median of seven cases per year, and the majority (57%) of cases occurred between January and April. Most (83%) cases were found on the island of Hawaii, with geographic information system (GIS) analysis identifying hot spots on the east side of the island. However, cases were identified on the other major islands as well, suggesting the risk of exposure is present statewide. Comparisons of cases from 2007 to 2017 with cases from previous assessments found no statistical differences in cerebrospinal fluid results, peripheral blood results, or ages of cases. However, differences in geographic distribution of the cases were statistically significant. Improved testing and increasing awareness of the disease have contributed to our efforts to better understand the general risk factors and modes of transmission present in Hawaii and also helped improve our prevention efforts, although we still do not fully understand the specific causes of cases being concentrated in certain parts of the state over others. Continued outreach efforts, including public forums and publication of preliminary clinical guidelines, aim to inform and improve our public health response and efforts to prevent angiostrongyliasis.

## INTRODUCTION

Angiostrongyliasis, also known as rat lungworm disease, is caused by the parasitic nematode *Angiostrongylus cantonensis.* Its primary host includes several species of rats, primarily those in the *Rattus* genus,^1–5^ in which mature *A. cantonensis* lay eggs. These eggs hatch into first-stage larvae, which are then expelled in the rat’s feces. Intermediate hosts, including snails and slugs, ingest the contaminated feces, and the first-stage larvae enter these hosts and develop into third-stage larvae. If a rat eats an infected intermediate host, the third-stage larvae infect the rat, in which they can continue to develop into mature adults, reproduce, and continue the cycle.

Human infections with *A. cantonensis* occur when individuals ingest third-stage larvae of the parasite. In humans, however, the third-stage larvae are not able to develop into their adult stage and, therefore, eventually die after migrating to the central nervous system. The immune system’s reaction to the dead parasites is responsible for most symptoms associated with angiostrongyliasis.^6^

The primary clinical presentation of angiostrongyliasis is eosinophilic meningitis (EM). Common symptoms include headache, stiff neck, paresthesias, vomiting, and nausea; face or limb paralysis, photophobia, and disturbed vision can sometimes present as well.^7,8^ Uncommonly, in severe cases, high intracranial pressure caused by the infection can result in unconsciousness, coma, and sometimes even death.^7,8^ Signs and symptoms generally reflect those areas damaged by the migrating larvae and resulting inflammation. Treatment is mainly supportive; lumbar punctures, analgesics, and especially corticosteroids may be used to treat some of the associated symptoms.^8^ The use of anthelmintic drugs has been controversial with unclear benefits.^8–10^

Traditionally, since the first human *Angiostrongylus* infection was identified in 1945 in Taiwan,^11^ infections have been most commonly identified in Southeast Asia and the Pacific basin. However, with increased globalism, this parasite has continued to spread to other parts of the world, including to the Americas,^4,12–14^ with cases often identified in travelers returning from regions where angiostrongyliasis is endemic.^15–18^ In the United States, angiostrongyliasis has been present in Hawaii since at least 1959.^19,20^ However, recently, *A. cantonensis* has been found in both mollusk and rat hosts in the Gulf Coast region of the continental United States,^21–23^ and sporadic autochthonous cases have been identified in other areas as well.^24,25^ This suggests the range of the parasite continues to expand, and cases may continue to appear in regions previously unaffected.

In Hawaii, angiostrongyliasis is endemic and has been reportable to the Hawaii Department of Health (HDOH) since 2007. Two previous assessments, from 1959 to 1965 and from 2001 to 2005,^20,26,27^ have examined cases of EM related to angiostrongyliasis in Hawaii. We report updated findings on the number and description of angiostrongyliasis cases in Hawaii from 2007 to 2017.

## METHODS

### Case definition.

A probable case was defined as an individual who had clinical signs of angiostrongyliasis and supporting laboratory evidence of EM without any other possible causes of EM identified. Clinical signs of angiostrongyliasis were defined as having two or more of the following signs and symptoms: headache, neck stiffness or nuchal rigidity, visual disturbance, photophobia or hyperacusis, cranial nerve abnormality, abnormal skin sensation, sensory deficit, nausea or vomiting, documented fever, increased irritability if age less than 4 years, or bulging fontanelle if age less than 18 months. Eosinophilic meningitis was defined as having a cerebrospinal fluid (CSF) specimen with ≥ 6 leukocytes per mm,^3^ and eosinophil percentage (of leukocyte count) of ≥ 10% or absolute eosinophil count ≥ 10. A case was considered confirmed if they met the criteria for a probable case and either *A. cantonensis* larvae or young adult worms were identified in their CSF, had a positive real-time polymerase chain reaction (RTi-PCR) test for *A. cantonensis* DNA from a CSF specimen, or were epidemiologically linked to a confirmed case. Real-time polymerase chain reaction was not included in the case definition for confirmed cases until 2016 when the test was validated^28^; however, for the purposes of this study, we applied the definition retrospectively to cases who had positive RTi-PCR results before 2016.

### Descriptive epidemiology.

We reviewed HDOH angiostrongyliasis case evaluation forms and patient medical records for cases reported to HDOH from 2007 through 2017. The evaluation forms were completed during patient interviews conducted as part of the original investigations. They included demographic information, reported initial signs and symptoms, and exposure histories. Medical records were reviewed to collect clinical information, including reported symptoms, hospitalization status, and laboratory results. To assess and describe additional exposures and risk factors, a supplemental questionnaire was created and administered to cases. The questionnaire collected data about their residential settings (or rental property for non-resident cases), animals or pests present on property, food purchasing and cleaning practices, and power and water sources at their residence/property.

Cases’ likely exposure locations were mapped using ArcMap 10.3 (Esri, Redlands, CA). If a case’s likely exposure was known, the location of exposure was mapped; otherwise, home addresses were used for Hawaii residents, and the addresses where cases stayed while in Hawaii were used for non-residents. Relationships between cases and environmental factors (mean annual rainfall, mean surface temperature, and fractional vegetation cover^29,30^) at the census block group level were analyzed. Spatial autocorrelation and hot spot analysis of cases, which identifies regions having high rates or numbers of cases surrounded by other regions with high rates or numbers of cases (i.e., where high number of cases cluster spatially), was conducted at the census block group level.

### Comparison with past cases.

Case laboratory results (including CSF white and red blood cell counts, eosinophils, glucose and protein levels, and peripheral blood white blood cell count and eosinophil levels) and case demographics (including age, gender, and geographic distribution) were compared with cases previously reported from 2001 to 2005.^26,27^

### Statistical analysis.

Statistical analysis of the data was performed using R (R Foundation for Statistical Computing, Vienna, Austria). The Mann–Kendall test for monotonic trend was used to identify trends in annual case counts, the chi-squared test was used to compare the racial distribution of cases with Hawaii State’s racial distribution and to compare the geographic distribution of cases reported here with previously reported cases; the Welch’s t-test was used to compare cases reported between 2007 and 2017 with previously reported cases. Spearman’s rank correlation coefficient was used to explore the relationship between cases and environmental factors (mean annual rainfall, mean surface temperature, and fractional vegetation cover^29,30^). GIS analysis was conducted with ArcGIS 10.3 (Esri). Global Moran’s I was used to test spatial autocorrelation of cases, and Getis-Ord Gi* was used for hot spot analysis. Results with a *P*-value of < 0.05 were considered statistically significant.

## RESULTS

### Descriptive epidemiology.

**Figure 1.**
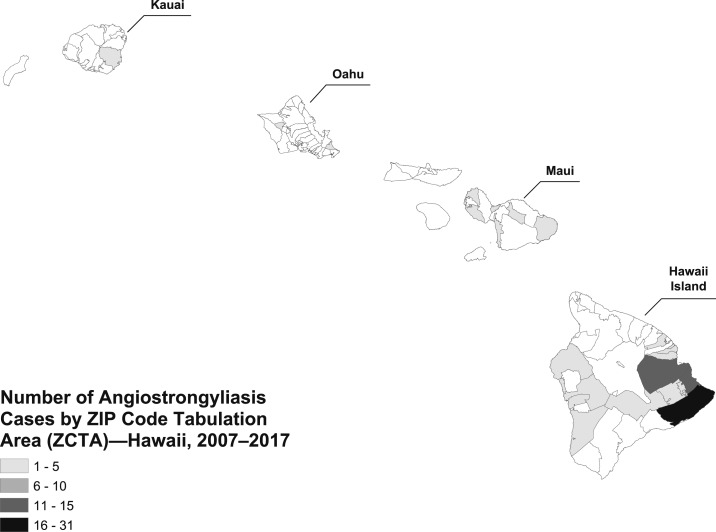
Angiostrongyliasis cases by ZIP code tabulation areas, Hawaii 2007–2017.

From 2007 to 2017, a total of 82 cases of angiostrongyliasis were identified in Hawaii, 51 (62%) confirmed and 31 (38%) probable. The greatest number of cases, 68 (83%), were reported on the island of Hawaii; 10 (12%) cases were identified on Maui, two (2%) on Kauai, and two (2%) on Oahu (Table 1, Figure 1). More than half of the cases, 51 (62%), were male, and the median age was 33 years (range 9 months–82 years). Majority of the cases were White (Table 1). Excluding the five non-Hawaii state residents, the racial distribution was significantly different than the racial distribution of the population of the State of Hawaii, with a much larger majority of the cases being White (60% versus 25%, *P* < 0.05). This difference remained significant when comparing pediatric and adult cases separately (Table 2).

**Table 1:** Angiostrongyliasis case demographics, Hawaii, 2007–2017

	No. (%) of cases
Age (*n* = 82)	Median 33 years (range 9 months–82 years)
Less than 10 years	12 (15)
10 to 17 years	3 (4)
18 years or older	67 (82)
Gender (*n* = 82)	
Male	51 (62)
Female	31 (38)
Race (*n* = 82)	
White	50 (61)
Native Hawaiian or Pacific Islander	14 (17)
Asian	5 (6)
Black, African American	1 (1)
Two or more	1 (1)
American Indian and Alaska Native	0 (0)
Unknown	11 (13)
County of residence* (*n* = 82)	
Hawaii	68 (83)
Maui	10 (12)
Kauai	2 (2)
Honolulu	2 (2)
Residency (*n* = 82)	
Hawaii State resident	77 (94)
Out-of-state	5 (6)
Case Status (*n* = 82)	
Confirmed	51 (62)
Probable	31 (38)

* For non-Hawaii residents, county in which they stayed while in Hawaii.

**Table 2:** Angiostrongyliasis case demographics for Hawaii-resident cases only, 2007–2017

	No. (%) of cases	Estimated mean annual incidence rate (per 100,000)*	
County of residence (*n* = 77)			
Hawaii	66 (86)	3.18	
Maui	8 (10)	0.46	
Honolulu	2 (3)	0.02	
Kauai	1 (1)	0.13	
Age (*n* = 77)	Median 33 years (range 9 months–82 years)		
Less than 10 years	12 (16)	0.62	
10 to 17 years	3 (4)	0.21	
18 years or older	62 (81)	0.52	
Gender (*n* = 77)			
Male	47 (61)	0.61	
Female	30 (39)	0.39	
Race (*n* = 77)		State of Hawaii†	
White	46 (60)	26%	*P* < 0.05
Native Hawaiian or Pacific Islander	14 (18)	10%	
Asian	5 (6)	38%	
Black, African American	1 (1)	2%	
Two or more	1 (1)	23%	
American Indian and Alaska Native	0 (0)	0%	
Others	0 (0)	1%	
Unknown	10 (13)	–	

* Number of cases divided by 11 years (2007–2017) divided by specified subgroup population obtained from 2012 Census Population Estimates, multiplied by 100,000.

† 2012 Census Population Estimates.

**Figure 2.**
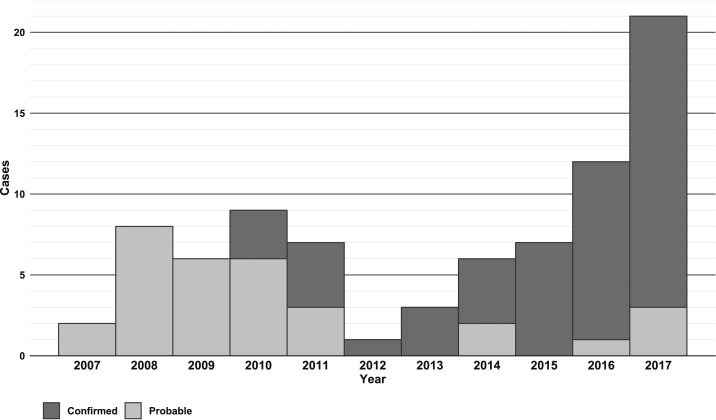
Angiostrongyliasis cases by year, Hawaii 2007–2017.

**Figure 3.**
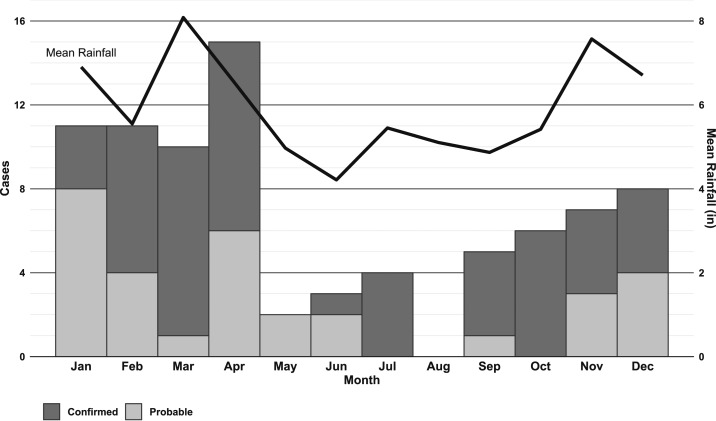
Angiostrongyliasis cases by month of onset and mean monthly rainfall, Hawaii 2007–2017.

The number of cases per year ranged from 1 to 21, median 7 (Figure 2). No monotonic trend was identified in the annual number of cases reported between 2007 and 2017 (*P* > 0.05). The median number of cases per month, after aggregating cases by month of onset over the assessment period, was 7 (range 0–15) with April having the most cases and August having the least (i.e., no reported cases). Majority of cases (57%) were reported between January and April (Figure 3). The mean annual incidence rate for 2007 to 2017 was highest on Hawaii Island (Table 2).

The most commonly reported symptoms for cases aged 10 years and older (70, 85%) were headache (83%), arthralgias/myalgias/body aches (74%), painful/sensitive skin (73%), and stiff neck (63%). For cases younger than 10 years (12, 15%), the most common symptoms were fever (75%), vomiting (67%), irritability (67%), fatigue (58%), and loss of appetite (58%).

The median amount of time between symptom onset and the initial lumbar puncture to obtain a CSF sample was 14 days (range 0–37 days). The median maximum eosinophil percentage in CSF specimens for cases was 42% (range 2–91%), whereas the median maximum eosinophil percentage in peripheral blood specimens was significantly lower (*P* < 0.05) at 14% (range 0–39%). Protein level of CSF specimens was generally elevated with a median maximum protein level of 116 mg/dL (range 45–357 mg/dL). There was no statistically significant difference between cases less than age 9 years and cases 10 years and older in CSF eosinophil levels, although cases less than 9 years had statistically significant lower CSF protein levels (77 mg/dL versus 137 mg/dL, *P* < 0.05). A total of 65 (79%) of the cases were hospitalized, with a median length of stay of 5 days (range 1–43 days). There were two (2%) deaths.

One (1%) case (pediatric) had *A. cantonensis* worms visible in their CSF sample, and 48 (59%) had positive PCR results (Table 3). Two cases whose initial CSF specimens were PCR negative (specimens collected 13 and 6 days after illness onset) had subsequent CSF specimens (9 and 16 days after the initial CSF collection, respectively) that were PCR positive. Cerebrospinal fluid eosinophil percentages increased for both cases between the PCR-negative specimens and PCR-positive specimens (increasing from 13% to 56% in the case with nine days between CSF specimens and 55% to 62% for the case with 16 days).

**Table 3:** Clinical and laboratory characteristics of angiostrongyliasis cases, Hawaii, 2007–2017

Days between onset and first LP (*n* = 79)	Median 14 days (range 0–37)
Maximum peripheral Eos (*n* = 77)	Median 14% (range 0–39%)
Maximum CSF Eos (*n* = 79)	Median 42% (range 2–91%)
Minimum CSF glucose (*n* = 70)	Median 41 mg/dL (range 15–117 mg/dL)
Maximum CSF protein (*n* = 68)	Median 116 mg/dL (range 45–357 mg/dL)
Hospitalized (*n* = 82)	65 (79)
Length of stay	Median 5 days (range 1–43 days)
Deaths (*n* = 82)	2 (2)
Polymerase chain reaction results (*n* = 56)	
Positive	48 (86)
Negative	8 (14)

Reported symptoms	No. (%) of cases
Ages 0–9 years (*n* = 12)	
Fever	9 (75)
Vomiting	8 (67)
Irritable	8 (67)
Fatigue	7 (58)
Loss of appetite	7 (58)
Encephalitis	6 (50)
Diarrhea	5 (42)
Stiff neck	4 (33)
Chills	4 (33)
Paralysis/weakness	4 (33)
Sensitive skin	3 (25)
Nausea	3 (25)
Headache	2 (17)
Abdominal pain	2 (17)
Altered mental status	2 (17)
Arthralgias/myalgias	1 (8)
Loss of consciousness	1 (8)
Rash	1 (8)
Cranial nerve abnormality	1 (8)
Bulging fontanelle	1 (8)
Ages 10 years and older (*n* = 70)	
Headache	58 (83)
Myalgias/arthralgias	52 (74)
Sensitive skin	51 (73)
Stiff neck	44 (63)
Nausea	36 (51)
Paralysis/weakness	36 (51)
Numbness	33 (47)
Fatigue	31 (44)
Fever	31 (44)
Chills	30 (43)
Loss of appetite	29 (41)
Abdominal pain	29 (41)
Photophobia	27 (39)
Vomiting	24 (34)
Disturbed vision	21 (30)
Encephalitis	15 (21)
Altered mental status	13 (19)
Constipation	12 (17)
Rash	10 (14)
Dizziness	7 (10)
Diarrhea	6 (9)
Loss of consciousness	3 (4)
Cranial nerve abnormality	3 (4)

CSF = cerebrospinal fluid; LP = lumbar puncture.

The extended questionnaire was completed by a total of 31 (38%) cases, all of whom were Hawaii residents. Four of every five cases (24, 80%) had observed snails or slugs on their property, and two-thirds (18, 67%) of cases reported observing rats or rat droppings. Most cases obtained their produce from farmer’s markets (18, 60%) and from commercial grocery stores (18, 60%). Fifteen (52%) of the cases grew their own food on their property. All cases reported storing food indoors with only two (7%) reporting also storing food outdoors; most, 26 (90%), used refrigerated storage. Half (50%) reported using a combination of sealed and loose storage, 11 (42%) reported sealed storage only, and two (8%) reported using loose storage only. When asked if they ever ate unwashed produce, 18 (64%) reported they did. Regarding utilities on cases’ properties, more than half (16, 55%) used a water catchment system on their property, whereas the remaining 13 (45%) were connected to municipal or county water systems. Two-thirds (19, 66%) of the cases’ only source of power was from the Hawaiian Electric Company, five (17%) only used solar power, two (7%) used both, and two (7%) reported not having a source of power (Table 4).

**Table 4:** Supplemental angiostrongyliasis questionnaire responses for cases in Hawaii from 2007 to 2017

	No. (%) of cases
Residency (*n* = 31)	
Hawaii State resident	31 (100)
Is the dwelling permitted or non-permitted? (*n* = 30)	
Permitted	26 (87)
Non-permitted	4 (13)
Is the dwelling open or enclosed? (*n* = 28)	
Enclosed	23 (82)
Open	5 (18)
What type of lavatory does the dwelling have? *(n* = 30)	
County sewage	11 (37)
Septic tank	7 (23)
Cesspool	5 (17)
Pit	3 (10)
Composting	1 (3)
Others/unknown	3 (10)
How is garbage disposed of? (*n* = 25)	
Off of their property (transfer station, etc.)	18 (72)
Composted	5 (20)
Both composted and off of property	2 (8)
Have they observed snails or slugs on the property? (*n* = 30)	
Yes	24 (80)
No	5 (17)
Does not recall	1 (3)
Have they observed rats or rat droppings on the property? (*n* = 27)	
Have observed rats and/or droppings	18 (67)
Have not observed rats and/or droppings	9 (33)
Do they keep any pets on the property? (*n* = 28)	
Yes	17 (61)
No	11 (39)
Do they have refrigerated food storage? (*n* = 29)	
Refrigerated storage	26 (90)
No refrigerated storage	3 (10)
Is their food stored indoors or outdoors? (*n* = 30)	
Indoors	28 (93)
Both indoors and outdoors	2 (7)
Is the food stored in sealed containers/areas or stored loose? (*n* = 26)	
Both sealed and loose containers/areas	13 (50)
Sealed containers/areas	11 (42)
Loose	2 (8)
Do they ever eat unwashed produce? (*n* = 28)	
Yes	18 (64)
No	10 (36)
How often do they wash their produce before use? (*n* = 27)	
Always	10 (37)
Often	5 (19)
Sometimes	7 (26)
Rarely	3 (11)
Never	2 (7)
What type of water is used to wash the produce? (*n* = 28)	
Catchment/rain water	15 (54)
County water	11 (39)
Others	2 (7)
Where do they obtain their produce?*(*n* = 30)	
Farmer’s markets	18 (60)
Grocery stores	18 (60)
Home-grown	5 (17)
Do they buy prewashed produce? (*n* = 28)	
No	16 (57)
Yes	4 (14)
Does not recall	8 (29)
Where is food prepared? (*n* = 30)	
Indoors	24 (80)
Outdoors	3 (10)
Both indoors and outdoors	3 (10)
Is any food grown on the property? (*n* = 29)	
Yes	15 (52)
No	14 (48)
What water sources are used on the property?*(*n* = 29)	
Catchment system	16 (55)
Municipal or county water	13 (45)
Other	3 (10)
What power sources are used on the property? (*n* = 29)	
Hawaiian Electric Light Company (HELCO)	19 (66)
Solar power	5 (17)
HELCO and solar power	2 (7)
Gas generators	1 (3)
None	2 (7)

* Categories are not mutually exclusive.

**Figure 4.**
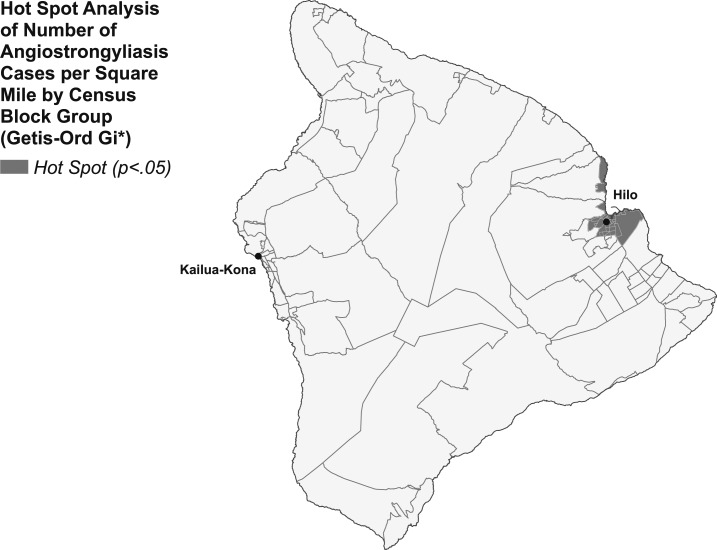
Hot spot analysis of number of cases per square mile by census block group, Hawaii Island 2007–2017.

**Figure 5.**
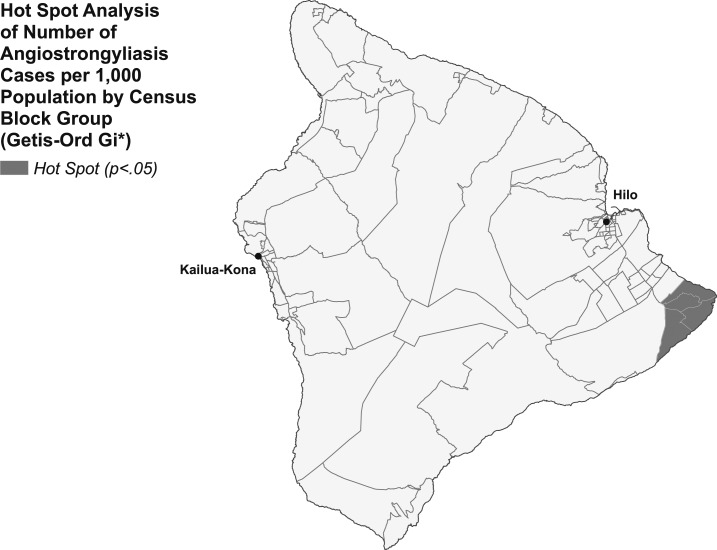
Hot spot analysis of number of Hawaii resident cases per 1,000 population by census block group, Hawaii Island 2007–2017.

Spatial clustering of cases was not identified for the islands of Kauai, Oahu, and Maui (Global Moran’s I *P* > 0.05), but was identified on Hawaii Island (Global Moran’s I *P* < 0.05). Hot spot analysis of number of cases per square mile by census block group level identified a hot spot centered around the city of Hilo on the east side of Hawaii Island (Figure 4). Analysis of cases among Hawaii residents per 1,000 population also identified a hot spot on the east side of the island, but southeast of Hilo, in a more rural region (Figure 5).

A statistically significant weak positive correlation between number of cases aggregated over the assessment period and mean annual rainfall and fractional vegetation cover was identified at the census block group level (*P* < 0.05, Table 5). A statistically significant weak negative correlation between number of cases and mean air temperature was also identified (*P* < 0.05, Table 5).

**Table 5:** Correlation of number of angiostrongyliasis cases aggregated over the assessment period and environmental factors at census block group level, Hawaii 2007–2017^28,29^

	Spearman’s rank correlation coefficient	*P*-value
Number of angiostrongyliasis cases vs.		
Mean annual rainfall	0.25	< 0.05
Mean annual air temperature	−0.23	< 0.05
Fractional vegetation cover	0.22	< 0.05

### Comparison with past cases.

Eighteen individuals with EM attributed to angiostrongyliasis between 2003 and 2005 in Hawaii were described in Hochberg et al.^27^ Comparing CSF results (white and red blood cells, eosinophil, glucose, and protein), peripheral blood results (white blood cells and eosinophils), and ages of the cases described here with the previously reported cases, we found no statistically significant differences (*P* > 0.05). The geographic distribution (by island) of cases reported between 2007 and 2017 compared with cases reported in Hochberg et al.^26^ was significantly different (*P* < 0.05), with the previously reported cases having a lower proportion of cases on Hawaii Island.

## DISCUSSION

Since angiostrongyliasis became reportable in the State of Hawaii in 2007, most cases have been reported from the Island of Hawaii, with hot spots identified on its east side. However, cases have also been identified on all the other major islands, suggesting the risk for infection exists statewide and is not restricted to Hawaii Island.

Cases occurred most frequently from January through April, which coincides with the months with the highest annual rainfall in Hawaii.^29^ This temporal association of cases with months with higher rainfall, along with the identified geographic correlation of cases with regions with higher rainfall, lower temperatures, and higher levels of vegetative cover could be representative of where human activity overlaps with the habitat of *A. cantonensis.* A recent article described a habitat model for the parasite, demonstrating a greater suitability to areas with increased precipitation and temperatures^31^ as well as its intermediate hosts (e.g., mollusks), implying an increased risk of exposure.

**Figure 6.**
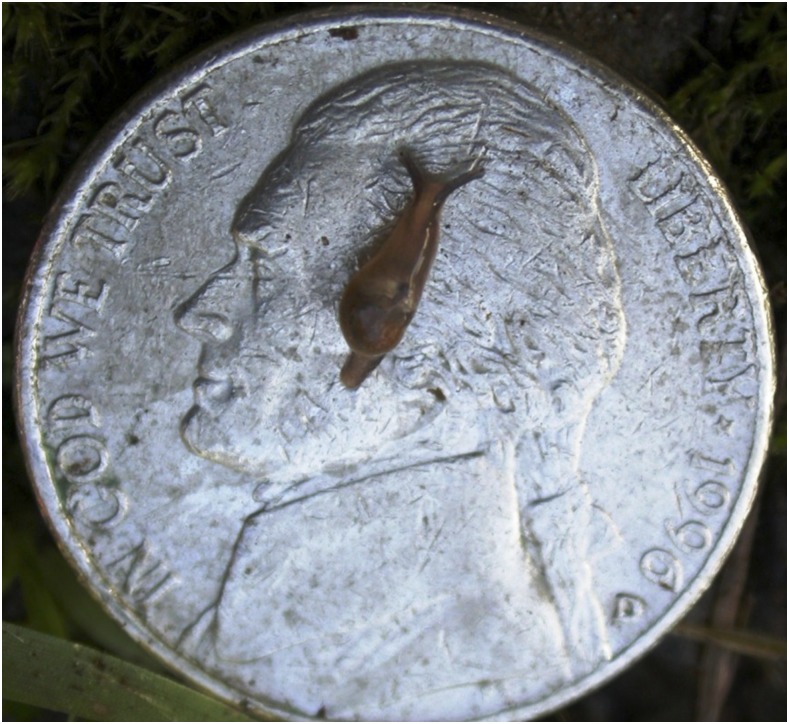
A juvenile *Parmaion* cf. *martensi* semi-slug on a nickel (credit: Hawaii Department of Health). This figure appears in color at www.ajtmh.org.

The intermediate hosts carrying the infective stage of the parasite have been identified statewide. One potentially important intermediate host, the invasive semi-slug *Parmarion* cf. *martensi*, was first identified on the island of Oahu in 1996, and then later on Hawaii Island in 2004, and most recently on Maui in 2017.^32,33^ This semi-slug has been shown to climb more frequently than other slug and semi-slug species and is attracted to rich food sources such as bird food, dog food, fruits, etc.^32^ They have also been shown to harbor high concentrations of the parasite in their tissue compared with other intermediate hosts.^34^ The potentially high concentration of parasite in this host and their behavior may be contributing to an increased risk of exposure to *A. cantonensis* in areas where the semi-slug is found. The size of young snails (neonates as small as 2 mm have been reported; Figure 6).^32^ could also contribute to the risk of unintentional ingestion. However, they are not the only host posing an exposure risk for infection. Many other *A. cantonensis* intermediate hosts exist in Hawaii as well, including the Cuban slug, *Veronicella cubensis*, the giant African snail, *Achatina fulica*, and the marsh slug, *Deroceras laeve*. These other slugs and snails are also often found in abundance in other environments (e.g., farms and home gardens) that could pose risk to humans.^32,34^

The primary source of infection in humans is the ingestion of raw or undercooked intermediate hosts.^7^ Although for some cases, the source of exposure was identifiable, such as a case who ate a slug on a dare or a cluster of cases who found slugs at the bottom of a serving bowl after drinking the contents, for many cases, the specific exposure event is never identified. People’s food habits and practices, including food preparation and storage, likely play a role in their exposure to the parasite. Many cases reported eating unwashed produce at least some of the time, which could increase exposure risk. Other habits and activities reported by many cases, such as growing food at home, purchasing produce at local farmer’s markets, or keeping pets (whose food, if not stored properly, could attract snails/slugs) on their property, could also potentially increase risk of exposure or could be correlated with high risk activities that have not been identified. It is an ongoing effort to better identify and describe these risk factors and the precise modes of transmission present in Hawaii. Adding to the difficulty is that although ingestion is the general recognized source of infection, the specific routes of ingestion, can vary greatly depending on location, culture, and other factors. This has been recognized since the first cases of angiostrongyliasis were identified in Hawaii, as Rosen reported in his initial assessment.^20^ Mapping and spatial analysis techniques such as GIS hot spot analysis, although enabling identifying particular areas of risk in the state and other geographic patterns to help better focus public health efforts, also clearly demonstrate the geographically shifting risk areas over time.

Until relatively recently, the only way to confirm cases of angiostrongyliasis was to identify the parasite in a case’s CSF specimen, which occurs very rarely; only one case between 2007 and 2017 was confirmed by this method. In collaboration between the HDOH State Laboratories Division (SLD) and the CDC, CDC developed an RTi-PCR test to detect *A. cantonensis* DNA in CSF specimens; this test can be performed at both SLD and CDC.^28^ In our experience, some probable cases may have initially negative RTi-PCR results. For that reason, if a case has a compelling clinical and epidemiologic history strongly suggesting angiostrongyliasis but has a negative RTi-PCR result, we recommend the RTi-PCR test be repeated on a subsequent CSF specimen obtained at least a week after the initial specimen. Still, angiostrongyliasis poses a diagnostic challenge, given various potential challenges (e.g., reluctance to undergo lumbar puncture, health-care access issues, and lack of recognition of the condition) with obtaining CSF from patients. There remain no available serological tests sufficiently reliable for clinical diagnosis; they often have issues with sensitivity and specificity as well as cross-reactivity with antibodies generated in response to other common helminths.^35^

The number of cases reported to HDOH is likely an underestimate of the actual number of cases in Hawaii. Individuals who are asymptomatic or have only mild symptoms are not likely to seek care and, therefore, will not be identified as cases and may not have laboratory findings to even help identify them. Our case investigations have helped better describe the risk factors associated with infection, but there are limitations. Given the length of time covered by the questionnaire, up to 30 days before illness onset, cases may have difficulty accurately recalling details about potential exposures. In addition, the strength of our conclusions is constrained by having had less than half of the cases complete the supplemental questionnaire. We are not able to determine what causes cases to be concentrated in the particular identified area of the state or why certain areas appear to have higher rates of infection than others, as many of the general risk factors for angiostrongyliasis are present statewide. However, improved testing and increased awareness of the disease are contributing to improved identification of new cases, which continue to add to our limited data. In addition, with increased awareness about the disease among health-care providers as well as the residents of Hawaii and visitors to the state, we anticipate a greater likelihood now to recognize cases which previously might have been missed.

With the understanding that the primary method of infection is ingestion of infected intermediate hosts, the most effective and feasible prevention measure at this time is public health education regarding how the disease is transmitted and best practices for food storage, hygiene, and preparation. In Hawaii, educational sessions have been held in communities to provide information on the disease and a forum where the public can receive answers to their questions. Our education efforts have also targeted health-care provider awareness with the formation of a clinical workgroup of the Hawaii Governor’s Joint Task Force on Rat Lungworm Disease and publication of preliminary clinical management guidelines^36^ to assure the timely recognition and reporting of cases to public health. These outreach efforts and our increasing understanding of the risk factors for angiostrongyliasis continue to inform and improve our public health response to the disease in Hawaii and may contribute to better understanding and preventing the disease elsewhere.

## Supplementary Material

Supplemental materials
