# Supplementary material for: Review of Cases of Angiostrongyliasis in Hawaii, 2007–2017
Source: Am J Trop Med Hyg. 2019 Jul 8;101(3):608–16. doi: 10.4269/ajtmh.19-0280 (PMC6726938; doi:10.4269/ajtmh.19-0280)
Supplement: Supplementary file 1 [file tpmd190280.SD1.pdf]

Supplementary Materials: *Illustrative Exposure Examples*

[Example 1 - Exposed food preparation areas]

Several cases' exposures may have been related to using food preparation areas exposed to the outdoors with greater likelihood of contamination by slugs/snails. One case's kitchen sink drained directly to the outside, and the case reported often observing snails/slugs crawling through it into the kitchen. The snails and slugs may have gotten into unsealed food and been consumed unknowingly. Another case reported keeping a blender in an outdoor kitchen area and frequently preparing smoothies. Snails/slugs may have crawled into the blender unnoticed and mixed into the smoothies.

[Example 2 - Shared kava bowl]

Six cases (3 confirmed, 3 probable) in 2017 were all linked to a single exposure, a shared bowl of kava (a plant-based drink common in the Pacific islands). They prepared the kava outdoors in two five-gallon buckets with water from an outdoor hose. After drinking the second bucket of kava, they discovered a slug at the bottom of the bowl from which they had been drinking. All six individuals had illness onset within two weeks of the exposure. Investigation of the property found slugs/snails inside the hose and covering the area where the hose was kept. The hose, in addition to being used for preparing kava, was also used as a water source for cooking. Other sources of slugs/snails were identified on the property including heavy vegetation, tarps, and detritus that provided conditions suitable for snails/slugs to thrive.

[Example 3 - Children putting things in mouth]

Cases of infection identified in young children were likely the result of normal developmental pica. One case, a one-year-old infant, often spent time crawling on a patio that the parents reported was frequently covered in snails/slugs. The child's parents did not observe them placing

a snail/slug in their mouth, but several weeks before onset they did have to remove an unknown substance from the child's mouth. Another case, a 10-month-old infant, frequently spent time in the garden with their mother where there were many opportunities for them to eat contaminated items directly from the plants or off the ground.

[Example 4 - Other known exposures]

For some cases specific, or at least likely, exposures were identified. One case became infected as the result of consuming a slug on a dare. Another was exposed after consuming a raw snail after gathering up dozens of them to be used to race against each other. A third case was exposed when they accidently ingested part of a snail when a weed-wacker they were using hit a snail, flinging pieces into their mouth.
